# Access to external credit during COVID-19: evidence from green SMEs in Italy

**DOI:** 10.1007/s11846-023-00654-9

**Published:** 2023-04-08

**Authors:** Maria Cristina Arcuri, Raoul Pisani

**Affiliations:** 1 Department of Economics and Management, University of Parma, Via J.F. Kennedy 6, 43125 Parma, Italy; 2 Department of Economics and Management, University of Trento, Via Inama 5, 38122 Trento, Italy

**Keywords:** COVID-19 pandemic, Green firms, SMEs, Bank credit, Trade credit, F64, G21, G32

## Abstract

This study explores the impact of being “green” as a response to variability in the business environment. We examine the financial resilience of green Small and Medium-sized Enterprises (SMEs) in Italy compared to non-green during the COVID-19 pandemic. We verify whether green SMEs are more able to attract external funding than non-green and whether green SMEs rely more heavily on trade credit than non-green ones. We carry out an analysis with 215,564 observations, of which 6844 refer to “green” firms, over the period 2017–2020 and we find that before and during the pandemic, Italian green SMEs do not attract more external funding than other SMEs, but they rely more on trade credit than non-green SMEs. Our results partially confirm the traditional substitution effect, and we suggest that the reasons for this relationship are also supplied in the literature which sees trade credit as a component of a long-term portfolio management strategy, i.e., as a tool for consolidating relationships with clients, for price discrimination and/or for increasing firm profitability in facing variable demand conditions. Our paper contributes to the literature in two ways. First, it investigates the relationship between the “green” characteristics of a firm and its level of economic and financial resilience during the pandemic. Second, it verifies whether, during a complex economic shock, green orientation increases or decreases the importance of trade credit relative to bank credit in financing the firm.

## Introduction

The impact of the COVID-19 pandemic was particularly strong, and on Small and Medium Enterprises (SMEs) stronger than that of the global financial crisis in 2008. In normal credit conditions, banks tend to consider SMEs as high-risk borrowers, because they are subject to liquidity shortfalls and bankruptcies reflecting conditions such as asymmetric information and agency problems, liquidity and profitability conditions and their ownership structure. Although relationship banking prevents errors in SME lending and in evaluating the creditworthiness of firms (Baas and Schrooten 2006), external shocks can severely disrupt this form of lending.

At the same time, especially since the 2008 crisis, the demand for a new economic and sustainable recovery has been recognized as increasingly important. European Union (EU) policy has placed great emphasis on a green economic growth (European Commission 2018, 2019). Greening the economy will have big impacts on many industries such as agriculture, transport, extractive industries, manufacturing, construction, and services, etc., in which SMEs prevail at local level. SMEs are in fact crucial for most national economies in the EU-28: they account for more than 99.8% of all businesses and generate more than 56% of value added and about 66.6% of employment.

In this context we explore the impact of being “green” for SMEs as a response to variability in the environment of corporate business. Because SMEs do not often have an ESG rating and because there are significant divergences in the ESG ratings published by six important rating agencies (Berg et al. 2022), we focus on “green” companies in industries characterized by a particularly high green component. We ask two main questions. First, did green SMEs have better access to external financial credit than non-green SMEs during the COVID-19 pandemic? Second, during the COVID-19 pandemic, did green SMEs rely more heavily on trade credit than banking and financial credit compared to non-green SMEs?

We concentrate on Italian SMEs, because in Italy the shares of employment and value added generated by SMEs are particularly high (78.1 and 66.9% respectively) compared to the EU average. We focus on manufacturing firms for two reasons. First, firms in manufacturing sectors have a greater concentration of tangible assets (e.g., higher liquidation value) and have better access to debt financing (both trade and bank credit). Secondly we wish to avoid industry-specific firms, such as farms, in agriculture, and industries which may not be sufficiently clearly defined, such as services. Our results if, from one side, appear to exclude the existence, during the COVID-19 pandemic, of a better access to financial credit for green SMEs than for non-green ones, from the other side, confirm a higher importance of trade credit for green SMEs than for non-green ones. The reasons of this relationship are probably complex and can only partially be explained by poor access to bank credit for marginal firms, since on average green SMEs seem to be more profitable than non-green ones. On the contrary, this relationship could also be explained according to the literature which sees trade credit as a component of a long-term portfolio management strategy, i.e., as a tool for consolidating relationships with clients, for price discrimination and/or for increasing firm profitability in variable demand conditions.

This study makes a significant contribution to the literature in two ways. First, it investigates the relationship between the “green” component and the degree of economic and financial resilience of Italian manufacturing SMEs during the COVID-19 pandemic. Second, it aims to verify whether an orientation to the green economy during a complex economic shock like COVID-19 increases or decreases the importance of trade credit relative to bank/financial credit in financing the firm.

The rest of the paper is structured as follows. Section 2 reviews the literature and describes the hypotheses. Section 3 and 4 describe our sample, the variables used, the methodologies we used in our analysis and the results. Section 5 concludes.

## Literature review and hypotheses development

The impact of the COVID-19 pandemic has been investigated in many countries (Kuckertz et al. 2020) and in many industries (Demirguc-Kunt et al. 2021; Donthu and Gustaffson 2020; García-Carbonell et al. 2021; OECD 2020). Although the global financial crisis in 2008 severely affected financial risk of SMEs (Cowling et al. 2018), the effects of COVID-19 on SMEs are thought to be even more severe (Baker et al. 2020; Bartik et al. 2020; International Trade Centre 2020; Howell et al. 2020). For example, social venture crowdfunding faced numerous difficulties during the crisis (Farhoud et al. 2021), start-ups and small businesses in China (Brown et al. 2020) and in England (Brown and Rocha 2020) underwent a big decline in equity investments in the first quarter of 2020 compared with same quarter in 2019. Although SMEs enjoy greater flexibility and adaptive capacities (Bartz and Winkler 2016; Battisti and Deakins 2012; Burns 2016; Gilmore et al. 2013) than bigger firms, at the same time they have a higher probability of failure than large and established firms (Berger and Udell 1998; Davidson and Gordon 2016; Doern et al. 2019; Herbane 2013, 2019; May and Lixl 2019; Beck and Demirguc-Kunt 2006; Juergensen et al. 2020), partly due to their ownership structure (Martin et al. 2019).

From a theoretical standpoint, the effect of environmental responsibility on firm financing during the COVID-19 pandemic can be explained in positive terms for two reasons. First, although authorities and academics use differing definitions of the green economy (Caprotti and Bailey 2014; UNEPP 2011),^1^ empirical investigations in EU have shown that providing solutions to environmental problems can effectively create new sources of growth (Kasztelan 2017; Lavrinenko et al 2019; Šipilova et al. 2017). The COVID-19 pandemic renewed the emphasis in the literature on the importance of green growth in terms of a synergic relationship between economic growth and the environment (Guerin and Suntheim 2021; Kang and Lee 2021; Mol and Sonnenfeld 2000; O'Callaghan and Murdock 2021). In this context, recent studies (D’Amato 2021; D’Amato and Korhonen 2021; Korhonen and Granberg 2020; Palahì et al. 2020; Taherzadeh 2021) focus on the integration of the green economy, and a circular economy and bioeconomy are recognized as essential to sustainable development policies. Second, there is evidence that greater environmental orientation may decrease the likelihood of negative events both at the firm level (Bouslah et al. 2018) and at the economic level, because such firms, enjoying established reputations as being environmentally responsible, are able to access financial resources more easily (Branco and Rodrigues 2006; Godfrey 2005; Zeidan et al. 2015). On the other hand, if financial institutions and markets do not adequately recognize environmentally responsible practices as an important intangible asset in lowering credit risk, firms may suffer competitive disadvantage because investment in environmentally responsible practices can be costly in the short term and lead to low profitability (Goss and Roberts 2011). The environmental performance of firms has been extensively examined by researchers and policymakers (Banerjee et al. 2019; Bragdon and Marlin 1972; Bruna and Nicolo 2020; Lahouel et al. 2020; Muhammad et al. 2015; Porter and Van der Linde 1995; Williamson et al. 2006). Although proactive environmental practices seem to imply low levels of risk (Godfrey et al. 2009; Muhammad et al. 2015), reductions in the cost of debt (Bauer and Hann 2010), easier access to financial markets (Jo and Na 2012), and better conditions on loans (Goss and Roberts 2011; Magnanelli and Izzo 2017; Sharfman and Fernando 2008), other studies demonstrate that financial risk incurred by socially and environmentally responsible firms is higher than other firms (Kiernan 2007; Seeger and Hipfel 2007), and in some cases investigations are inconclusive (Lee and Faff 2009). A recent study (Wellalage and Kumar 2021) on 3915 unlisted firms in developing countries indicates that firm-level environmental performance has a positive impact on the loan size for firms, particularly small firms. In general terms, firms that exhibit proactive environmental practices are expected to carry a lower level of risk (Godfrey et al. 2009), can access the financial market less easily (Farza et al. 2021; Jo and Na 2012), and have greater leverage (Sharfman and Fernando 2008). These differences are thought to be particularly marked for manufacturing firms because there is evidence that firms in manufacturing industries have better access to debt (banking and trade financing) than non-manufacturing firms. This may reflect the greater concentration of tangible assets (Van Der Wijst and Thurik 1993; Jordan et al. 1998) or lower information asymmetries (La Rocca et al. 2010) between manufacturing compared to non-manufacturing firms. Other research on trade credit finds that firms in traditional or manufacturing sectors obtain trade credit more easily than firms in non-manufacturing industries (Mian and Smith 1992; Psillaki and Eleftheriou 2015). These results are confirmed by additional research in Europe (Casey and O’Toole 2014) and Japan (Taketa and Udell 2007).

Although most of the studies outlined above investigate the relationship between environmental performance and financial risk in normal economic conditions, we argue on the basis of these findings that green SMEs are more likely to have been more financially resilient than non-green SMEs during the COVID-19 pandemic. Our first hypothesis is as follows:

### Hypothesis 1

Green SMEs show a higher proportion of external funding/total assets than non-green SMEs both during the COVID-19 pandemic and in normal times (where external funding is represented by trade credit + banking credit).

There is overwhelming evidence to support the positive effect of environmental performance on firm performance, but there is as yet little evidence on the impact of a financial crisis on the relationship between social performance and firm risk (Bouslah et al. 2018; Lins et al. 2017, 2019; Marsat et al. 2020). The relationship between environmental performance and financial risk during the COVID-19 pandemic has been recently investigated on a sample of 3356 MSMEs (micro and small-medium sized enterprises) located in southern and eastern Europe (Wellalage and Kumar 2020) and on a sample of 6597 SMEs located in eastern Europe (Wellalage et al. 2021). The findings suggest that green companies showed a lower probability of liquidity shortfall and bankruptcy during the pandemic, and also the existence of a significant and positive relationship between environmental orientation of the firm and access to external finance. Better environmental performance appears to reduce the level of idiosyncratic risk as perceived by stakeholders (Bouslah et al. 2018), increase the access to financial resources (Zeidan et al. 2015) and boost the restoration of stakeholder trust following periods of crisis (Pricewaterhouse Coopers 2013). These results are consistent with research on listed firms (Farza et al. 2021) before the COVID-19 pandemic: green investments enhance resource efficiency and corporate reputation with corresponding effects on financial performance. The theoretical explanation of the relationship between environmental and financial performance could be that a higher level of trust between firm and shareholders leads stakeholders to increase their level of collaboration and reciprocation and enhances the firm’s reputation (Lins et al. 2017, 2019). High trust levels also boost innovation, which is in turn a competitive advantage in times of crisis, allowing firms to resist and even flourish (Huang et al. 2020) and they provide greater advantages than protection against idiosyncratic firm-specific legal risks (Hong and Liskovich 2019). Higher levels of trust can also extend to supplier companies, which can take advantage of more detailed information on their green clients, including lower levels of risk than non-green clients. Thus, our next two hypotheses are as follows:

### Hypothesis 2a

Green SMEs rely on higher levels of trade credit than banking/financial credit both during the COVID-19 pandemic and in normal times.

### Hypothesis 2b

Green SMEs depend on higher levels of trade credit than non-green SMEs both during the COVID-19 pandemic and in normal times.

The literature on trade credit shows that its relationship with bank credit is complex (Mateut et al. 2006; Matias Gama and Van Auken 2015; McGuinness and Hogan 2014; Wilner 2007). It may appear that trade credit is a substitute for bank credit, because it is mainly requested by companies with poor access to bank credit (Fisman and Love 2003; Nilsen 2002) especially in cases of financial crisis (Love et al. 2007) during monetary restrictions (Brechling and Lipsey 1963; Duca 1986; Herbst 1974; Jaffee and Modigliani 1969; Jaffee 1971; Mateut 2005; Meltzer 1960; Wilner 2000) and in countries with a poorly developed local banking system (Alessandrini et al 2009; Benfratello et al. 2008; Bonaccorsi di Patti and Gobbi 2001; Gagliardi 2009; Guiso et al 2004; La Rocca et al 2010; Petersen and Rajan 1995). But while borrowers are likely to view bank and trade credit as substitutes, trade credit can be considered as complementary to financing by financial intermediaries, who lend to suppliers who in turn relend to their clients (Carbo-Valverde et al. 2016; Choi and Yungsan 2005; Cull et al 2009; Demirgüç-Kunt and Maksimovic 2001; García-Teruel and Martínez-Solano 2010; Garcia-Appendini and Montoriol-Garriga 2013; Jain 2001; Love et al 2007; McMillan and Woodruff 1999; Ogawa et al 2013; Tsuruta 2015). Recourse to trade credit can also be explained by a competitive advantage of supplier companies over banks in the exploitation of informal means that guarantee the repayment of the loan. This competitive advantage may derive from better and/or less expensive information on the financial situation of client firms (Biais and Gollier 1997; Petersen and Rajan 1997; Pike et al. 2005), monitoring advantages (Bukart and Ellingsen 2004; Emery 1987; Freixas 1993; Schwartz and Whitcomb 1979), and product market imperfections (Brennan et al 1988). In this context, some studies (Cannari et al. 2004; Ng et al. 1999) demonstrate big differences across industries in trade credit terms but little variation within industries across time, so that trade credit can be viewed as a component of a long-term portfolio management strategy (Emery 1987), a tool for consolidating relationships with clients reflecting guarantee of product quality (Deloof and Jegers 1996), for price discrimination (Bougheas et al. 2009; Lee and Stowe 1993; Long et al. 1993; Petersen and Rajan 1997; Schwartz and Whitcomb 1978, 1979), for increasing firm profitability (Martínez-Sola and García-Tereul 2014) and for dealing with variable demand conditions (Long et al. 1993). Since trade credit can be used to finance purchases (accounts payable) and clients (accounts receivable), studies examining the relationship between accounts payable and receivable conclude that there is sufficient evidence to support the matching hypothesis, which is that trade debt is influenced by trade credit policy (Bastos and Pindado 2013; Fabbri and Klapper 2008; García-Teruel and Martínez-Solano 2010; Mian and Smith 1994; Paul and Wilson 2007) and some evidence that accounts payable in one year depend on those of the previous year (Bussoli 2017). These results suggest that operational conditions, transaction costs and firm business environment influence the demand and supply of trade credit (Summer and Wilson 2000) and could explain persistent time invariant aspects of trade credit within industries and high heterogeneity across industries (Wilson and Summers 2002; Marotta 2005) in this case too particularly in cases of monetary tightening (Dedola and Lippi 2000; Guiso et al. 2000). Even the importance of trade credit in the different stages of the business life cycle of SMEs depends on the sector, which is extremely important in the financial decisions of SMEs (Psillaki and Eleftheriou 2015; Yazdanfar and Öhman 2017).

In this context, a recent empirical analysis (Arcuri and Pisani 2021) of Italian Medium-sized Enterprises (MEs) examines the relationship between trade credit and green-oriented firms. A panel analysis is applied to 101,250 observations over the period 2010–2019. The results show that, although trade credit is more important for younger, smaller, less profitable and less liquid MEs, green MEs rely more on trade credit than non-green MEs. They offer more trade credit to their clients and at the same time they receive more trade credit than non-green. In addition, green MEs tend to rely on trade credit, regardless of the stage of the company’s life cycle, more stably than non-green MEs. The closer appreciation and knowledge of green manufacturing by companies in same industry compared to the appreciation of green ME characteristics by banks and financial intermediaries may explain these results.

## Data, sample and methodology

### Data and sample

To answer our research questions, we download the entire records of Italian SMEs from the AIDA Bureau van Dijk database. We aim to cover the periods before and during the COVID-19 pandemic, so our sample includes all companies for which data are available for the period 2017–2020. Our final sample consists of 53,891 SMEs. Following European Commission^2^ definitions, a small enterprise has between 11 and 50 employees, between 2 and 10 million euro annual turnover, and between 2 and 10 million euro balance sheet total. A medium-sized enterprise has between 50 and 250 employees, between 10 million and 50-million euro annual turnover, and between 10 and 43 million euro balance sheet total. The SMEs included in our sample belong to an “industry in the strict sense”. In the ATECO 2007 code, the classification of economic activity used by ISTAT (the Italian National Institute of Statistics), these are found in Sections B-E. Section B is “Extraction of minerals from quarries and mines”, Section C is “Manufacturing activities”, and Sections D and E are “Supply of electricity, gas, steam and air conditioning” and “Supply of water; sewer networks, activities of waste management and remediation”.

We apply the Differences-in-Differences methodology (diff-in-diff) and carry out a panel analysis and OLS regressions with 215,564 observations, of which 6844 refer to firms belonging to the “green” sector. The concept of “green economy” (Barbier 2012) is perceived as a pathway to sustainability by international organizations such as The World Bank (2012), the United Nations Environment Programme (2011), and the European Commission (2018). A study by the Politecnico di Milano and Camera di Commercio di Milano (2012) identifies specific “green” sectors on the basis of the following concepts. First, the green economy has as its pillars resource efficiency and natural capital. And second, an inclusive green economy is associated with economic growth, human development and opportunities, for people—to improve the living environment and jobs—and for businesses—to increase benefits through more efficient production practices that generate savings. Following the study by the Politecnico di Milano and Camera di Commercio di Milano (2012), we select industries which have an important green component, among those in industry “in the strict sense”, using the following ATECO 2007 codes: Collection, Reuse, Recycling of Waste (Codes 38.11.00, 38.12.00, 38.21.01, 38.21.09, 38.22.00, 38.31.10, 38.32.10, 38.32.20, 38.32.30 and 39.00.01), Efficiency of water systems: (Code 36.00.00), Planning, Reclamation and Rehabilitation of the territory (Code 30.00.09), Waste water treatment (Code 37.00.00), Energy Storage (Code 27.20.00). Most of the companies included in the sample (89.97%) are companies at least 10 years old. Consistently with previous literature (Abu Bakar 2011; Ayyagari et al. 2011; Fort et al. 2012), we classify young firms as those of up to 5 years, mature firms from 6 to 10 years and old firms over 10 years. Panels A and B of Table 1 present the sample distribution by type of sector (i.e., green or non-green) and age (i.e., young, mature or old), respectively.

**Table 1:** Observations by type of sector and firm age

Type of sector	No. of observations	% of total
*Panel A: observations by type of firm sector*		
Green	6844	3.17
Non-green	208,720	96.83
Total	215,564	100
*Panel B: observations by firm age*		
Young	4588	2.13
Mature	17,032	7.90
Old	193,944	89.97
Total	215,564	100

In order to study differences between green and non-green SMEs in terms of economic and financial performance, the degree of financial resilience and the size, age of the firm and the orientation towards trade or bank credit before or during the COVID-19, we collect a set of information that includes age, sector and financial ratio for each SME. Our data source is the AIDA Bureau van Dijk database. Consistently with much existing literature (Canto-Cuevas et al. 2019; Cuñat 2007; Fukuda et al. 2007; García-Teruel and Martínez-Solano 2010; Petersen and Rajan 1997; Yang 2011), we use “Trade credit” (i.e., trade payable/total assets) and “Full credit” (i.e., trade credit + bank credit/total assets),^3^ as dependent variables. We observe the use of trade and full credit during the firm’s life cycle by considering among the independent variables “Age”, which is the number of years of a firm’s activity. In particular, we use the logarithm of (1 + age). We also consider firm “Size” (i.e., the logarithm of the total assets); “Profitability”, which is the ratio of Earnings Before Interest, Taxes, Depreciation and Amortization (EBITDA) to total assets; “Liquidity” or “current ratio” (i.e., short-term assets/short-term liabilities); trade credit in the previous year (“Trade credit_t-1_”), which is the ratio of trade payable to total assets of the previous year; “Account receivable” (i.e., trade receivable/total assets). In order to verify whether a potential substitution effect between trade and bank credit exists, we consider “Short-term bank credit” (following AIDA, its bank debts within 12 months, divided by total assets) and “Long-term bank credit” (following AIDA, its bank debts over 12 months, divided by total assets). Table 2 summarizes the descriptive statistics for the entire period 2017–2020. The descriptive statistics for the sub-periods 2017–2019 and 2020 are reported in Appendix (see Table 10).

**Table 2:** Descriptive statistics—2017–2020

Variables	Mean	Median	SD	Min	Max
Full credit	0.2728	0.2236	0.2943	0.000	0.998
Trade credit	0.1754	0.1557	0.1549	0.000	0.995
Age	29.536	28.000	15.899	2.000	142.000
Size	8.595	8.460	1.026	1.908	14.976
Profitability	0.077	0.072	0.151	− 4.533	0.998
Liquidity	1.487	1.110	1.255	0.000	10.000
Trade credit_*t* − 1_	0.1830	0.1639	0.1619	0.000	0.975
Account receivable	0.2469	0.2329	0.1900	0.000	0.959
Short-term bank credit	0.084	0.024	0.146	0.000	0.999
Long-term bank credit	0.070	0.012	0.096	0.000	0.988
Short-term bank credit_*t* − 1_	0.075	0.001	0.194	0.000	0.985
Long-term bank credit_*t* − 1_	0.053	0.001	0.174	0.000	0.981

Source: AIDA Bureau van Dijk

Table 3 presents the correlations between all the variables used in the analysis. We also run a test of Variance Inflation Factors (VIF). Table 4 shows that no explanatory variables show VIF values above 10, so multicollinearity is not a concern in our models (Yang et al. 2019).

**Table 3:** Correlation coefficient matrix

Variables	Full credit	Trade credit	Age	Size	Profitability	Liquidity	Trade credit_*t* − 1_	Account receivable	Short-term bank credit	Long-term bank credit
Full credit	1.000									
Trade credit	0.7234*	1.000								
Age	− 0.0555*	− 0.141*	1.000							
Size	0.1080*	− 0.056*	0.186*	1.000						
Profitability	− 0.1148*	− 0.032*	− 0.018*	0.103*	1.000					
Liquidity	− 0.3361*	− 0.241*	− 0.100	0.027*	0.196*	1.000				
Trade credit_*t* − 1_	0.5960*	0.817*	− 0.145*	− 0.063*	− 0.021*	0.208*	1.000			
Account receivable	0.3795*	0.488*	− 0.070*	− 0.009*	0.070*	0.023	0.434*	1.000		
Short-term bank credit	0.7210*	− 0.245*	− 0.029	− 0.070*	− 0.145*	− 0.296*	− 0.194*	0.189*	1.000	
Long-term bank credit	0.5665*	0.111*	0.011	0.099*	− 0.056*	− 0.126	0.110*	0.031*	0.184*	1.000

^*^Indicates significance at the 5% level

**Table 4:** VIF test

Variable	VIF	1/VIF
Age	1.09	0.9199
Size	1.08	0.9295
Profitability	1.08	0.9270
Liquidity	1.19	0.8416
Trade credit_*t* − 1_	1.34	0.7455
Account receivable	1.28	0.7807
Short-term bank credit	1.20	0.8354
Long-term bank credit	1.06	0.9458
Short-term bank credit_*t* − 1_	1.15	0.8711
Long-term bank credit_*t* − 1_	1.07	0.9360

### Methodology

To answer our research questions, we use an OLS regression to compare 2019 with 2020 and a panel data approach. We also compare the three-year period 2017–2019 with the year 2020 (see Appendix). We next apply the diff-in-diff methodology. The equation models include the variables described in Sect. 3.1 and additional time dummies as control variables:

1 $$\begin{aligned} {\text{Trade credit}}_{it} & = \beta_{0} + \beta_{{1}} {\text{Age}} + \beta_{{2}} {\text{Size}} + \beta_{{3}} {\text{Profitability}} \\ &\quad + \beta_{{4}} {\text{Liquidity}} + \beta_{{5}} {\text{Trade credit}}_{{t - {1}}} + \beta_{{6}} {\text{Account receivable}} \\ &\quad + \beta_{{7}} {\text{Short - term bank credit}} + \beta_{{8}} {\text{Long - term bank credit}} \\ & \quad + {\text{time dummies}} + \mu_{it} + \varepsilon_{it} \\ \end{aligned}$$

2 $$\begin{aligned} {\text{Full credit}}_{it} & = \beta_{0} + \beta_{{1}} {\text{Age}} + \beta_{{2}} {\text{Size}} + \beta_{{3}} {\text{Profitability}} \\& \quad + \beta_{{4}} {\text{Liquidity}} + \beta_{{5}} {\text{Trade credit}}_{{t - {1}}} \\ &\quad + \beta_{{6}} {\text{Account receivable}} + \beta_{{7}} {\text{Short - term bank credit}}_{{t - {1}}} \\ &\quad + \beta_{{8}} {\text{Long - term bank credit}}_{{t - {1}}} + {\text{time dummies}} + \mu_{it} + \varepsilon_{it} \\ \end{aligned}$$

where *i* is the firm, and *t* is the time period; *µ*_*i*_ represents the firm-specific effects, and *ε*_it_ represents the measurement errors. As specified in the previous section, “Trade credit” is the dependent variable of Eq. (1) while “Full credit” is the dependent variable of Eq. (2). Following previous studies (e.g., Canto-Cuevas et al. 2016, 2019), “Age” is transformed into the logarithm of (1 + age) in the equations. We estimate Eq. (1) using estimators of fixed and random effects to take the individual effects into account (*µ*_*i*_), with clustered standard errors at firm level. The Hausman test is performed to ascertain whether the individual effects are fixed or random. If the null hypothesis is rejected, correlation between the independent variables and the individual unobservable effects does not exist and the random effects model is considered a good estimator. The Hausman test results suggest that fixed effect models are appropriate for our dataset. Tables reported in Sect. 4, therefore, show only the results of estimation with a fixed effect model.

Diff-in-diff methodology makes it possible to estimate the effect of a "treatment" (i.e., COVID-19 pandemic) on subjects. It uses a “treatement group or treated” (green SMEs) relative to a control group not exposed to the treatment (non-green SMEs) (e.g., Bertrand et al. 2004). The two groups are observed in two periods, one before and one after the treatment (i.e., 2019 and 2020, respectively). Diff-in-diff in fact makes it possible to: (1) observe how the treatment group mean changes before and after the treatment; and (2) compare this change with the mean over time of a similar group which did not undergo the treatment (i.e., control group). The regression model used for the estimation is the following:

3 $$\begin{aligned} Y_{{{\text{i}},{\text{t}}}} & = \beta_{0} + \beta_{1*} {\text{Green}} + \beta_{2} *{\text{COVID}} \\ &\quad + \beta_{3} *{\text{Green}}*{\text{COVID}} + \beta_{i} *Z_{i} + \varepsilon_{it} \\ \end{aligned}$$

where *Y* is the outcome variables of interest (i.e., *Y* = Full credit; *Y* = Short-term bank credit; *Y* = Long-term bank credit; *Y* = Trade credit) of firm *i* at time *t*, observed in periods 1 and 2. “Green” is a dummy variable which takes the value of 1 for "treated" subjects (green SME) and 0 otherwise (non-green SME). In other words, the variable “Green” captures the possible a priori differences between the treated group and the control group. “COVID” is a dummy variable which takes value 1 in 2020 (the year of COVID-19 pandemic) and 0 otherwise. The coefficient of this variable tells us how much the external credit (full credit, trade credit, short-term bank credit and long-term bank credit) of the control group changes on average after treatment. The variable “Green*COVID” represents the interaction between “Green” and “COVID” and takes a value of 1 for subjects treated in the second period. The coefficient of the variable (Green*COVID) is the parameter of interest because it expresses the effect of the treatment, or the differential impact of COVID-19 on green SMEs. The diff-in-diff estimator is the difference between the mean differences: it takes the difference in the treatment group before and after the treatment (the treatment effect) and subtracts the difference in the control group before and after the treatment (the trend over time), as in the following formula:

4 $$\left( {{\text{Treatment}}\_{\text{post }} - {\text{ Treatment}}\_{\text{pre}}} \right) - \left( {{\text{Control}}\_{\text{post }} - {\text{ Control}}\_{\text{pre}}} \right) \, = {\text{ Diff - in - Diff estimate}}$$

“*Z*” in Eq. (3) refers to a set of controls for credit ratios, which are Age, Size, Profitability and Liquidity as measured in previous equations. We limit the number of controls, and do not regress credit ratios on other credit ratios (or on the lags of the credit ratios) in order to avoid significant autocorrelation of errors and inflated standard errors. $\varepsilon$_*it*_ indicates the error term.

## Results

### Green orientation and external credit during COVID-19

In order to investigate the relationship between the “green” component and the degree of economic and financial resilience during the COVID-19 pandemic, and to verify whether orientation to the green economy tends to increase or decrease the importance of trade credit relative to bank/financial credit in financing the firm, we carry out our analysis on green and non-green SME sub-samples. Table 5 shows the trend over time (2017–2020) of the mean values of the main variables examined. The first group (green SMEs) appear to be funded differently compared to the second (non-green SMEs). Although trade credit received (from suppliers) is higher than bank credit both for green and non-green firms, the importance of trade credit received is much higher for green companies, and it seems to act as a substitute for short term bank credit.

**Table 5:** Descriptive statistics green and non-green SMEs—trend of mean values (2017–2020)

Variables	Green				Non-green			
	2017	2018	2019	2020	2017	2018	2019	2020
	Mean				Mean			
Full credit	0.3002	0.2959	0.2800	0.2351	0.2942	0.2854	0.2734	0.2375
Trade credit	0.2072	0.2044	0.1947	0.1789	0.1883	0.1807	0.1735	0.1538
Profitability	0.0842	0.0832	0.0893	0.09471	0.0820	0.0811	0.0778	0.0645
Liquidity	1.5006	1.5239	1.5531	1.7527	1.3705	1.4125	1.4654	1.7119
Trade credit_*t* − 1_	0.2058	0.2073	0.2044	0.1947	0.1865	0.1882	0.1807	0.1735
Account receivable	0.2944	0.2964	0.2941	0.2728	0.2577	0.2533	0.2474	0.2206
Short-term bank credit	0.0699	0.0689	0.0634	0.0526	0.0921	0.0922	0.0897	0.0644
Long-term bank credit	0.0645	0.0679	0.0664	0.0855	0.0616	0.0632	0.0642	0.0955

Source: AIDA Bureau van Dijk

Table 6 summarizes descriptive statistics for green and non-green SMEs for 2019 and 2020. The descriptive statistics over the whole period 2017–2020 and the sub-periods 2017–2019 and 2020 are reported in the Appendix (Tables 11, 12).

**Table 6:** Descriptive statistics for green and non-green SMEs, 2019 in comparison with 2020

Variables	Green		Non-green			Green		Non-Green		
	2019					2020				
	Mean	Std.Err	Mean	SE	*t* Statistic	Mean	SE	Mean	SE	*t* Statistic
Full credit	0.2800	0.0067	0.2734	0.0013	0.9075	0.2351	0.0060	0.2375	0.0012	− 0.3638
Trade credit	0.1947	0.0045	0.1735	0.0007	5.2714***	0.1789	0.0044	0.1538	0.0007	6.4305***
Profitability	0.0894	0.0035	0.0778	0.0007	2.7848*	0.09471	0.0032	0.0645	0.0007	7.6590***
Liquidity	1.5531	0.0319	1.4654	0.0061	2.5995*	1.7527	0.0382	1.7119	0.0069	1.0287
Trade credit_*t* − 1_	0.2044	0.0047	0.1808	0.0007	5.6325***	0.1947	0.0045	0.1735	0.0007	5.2714***
Account receivable	0.2941	0.0059	0.2474	0.0009	9.3540***	0.2728	0.0057	0.2206	0.0008	10.6757***
Short-term bank credit	0.0634	0.0034	0.0897	0.0007	− 6.5393***	0.0526	0.0025	0.0644	0.0006	− 3.4356***
Long-term bank credit	0.0664	0.0028	0.0642	0.0006	0.6260	0.0855	0.0033	0.0955	0.0006	− 2.7726*

*p-value < 0.1

**p-value < 0.05

***p-value < 0.01

Source: AIDA Bureau van Dijk

Table 6 reports a period-by-period comparison between green and non-green SMEs. As might be imagined, the impact of COVID-19 is very significant for both green and non-green companies in terms of full credit, trade credit (accounts payable), accounts receivable and short-term credit decrease. Increases in long-term bank credit in 2020 can be explained by the introduction of special state guarantees on bank loans with maturity over 1 year made to companies during the pandemic,^4^ and in fact, the growth of bank loans other than short term loans to companies in 2020 is also certified by Bank of Italy statistics.^5^ Table 6 also shows that liquidity increases for both green and non-green companies. This is consistent with the fact that in 2020, when the pandemic started, there was an increase in liquid capital due to the increase in long-term banking debt, and the uncertainty and fears for the future which led companies to postpone investments. Table 6 also shows that profitability in 2020 for green companies increased compared to 2019, while there was a significant reduction for non-green companies. Green SMEs show higher profitability than non-green in both periods, and higher liquidity than non-green ones in normal times.

In order to deepen the analysis on changes in levels of trade credit as a component of full credit, as well as short-term bank credit and long-term bank credit, we also apply diff-in-diff methodology. Table 7 shows the results (Table 13 in the Appendix reports the results considering 2017–2019 as the period before the treatment, i.e., 2020).

**Table 7:** Results of diff-in-diff analysis (dependent variables: full credit, Trade credit, Short-term bank credit, long-term bank credit)

	Dependent variable (outcome variables)			
	Full credit	Trade credit	Short-term bank credit	Long-term bank credit
Constant	0.2307***	0.2056***	0.0326***	− 0.0094**
	(0.0087)	(0.0046)	(0.0043)	(0.0041)
Green	− 0.0023	0.01706***	− 0.0221***	0.0033
	(0.0070)	(0.0037)	(0.0035)	(0.0033)
COVID	− 0.0036*	− 0.0148***	− 0.0213***	0.0331***
	(0.0019)	(0.0009)	(0.0009)	(0.0009)
Green*COVID	0.0048	0.0019***	0.0148**	− 0.0128**
	(0.0103)	(0.0005)	(0.0052)	(0.0048)
Age	− 0.0218***	− 0.0311***	0.0086***	0.0001
	(0.0015)	(0.0008)	(0.0008)	(0.0007)
Size	0.0327***	0.0128***	0.0091***	0.0110***
	(0.0009)	(0.0005)	(0.0005)	(0.0004)
Profitability	− 0.1524***	− 0.0002	− 0.1010***	− 0.0328***
	(0.0063)	(0.0034)	(0.0032)	(0.0030)
Liquidity	− 0.0664***	− 0.0260***	− 0.0277***	− 0.0127***
	(0.0007)	(0.0004)	(0.0003)	(0.0003)
N. observations	84,564	84,465	84,556	84,564
*R* squared	0.0429	0.0882	0.1020	0.0429

Robust standard errors are shown in brackets

*p-value < 0.1

**p-value < 0.05

***p-value < 0.01

Results presented in Table 7 confirm that there is a differential impact of COVID-19 on green SMEs, probably due to policy changes in green firms compared to non-green firms during the peak period of the pandemic (2020). We find that COVID-19 significantly reduced the difference in long-term bank credit (-0.0128 basis points). On the other hand, COVID-19 increased the difference in trade credit and short-term bank credit of green SMEs (0.0019 and 0.0148 basis points, respectively). After COVID-19, in fact, the levels of both trade credit and short-term bank credit fell, but for green SMEs less sharply than for non-green ones.

### Green orientation and determinats of trade credit during COVID-19

Tables 6 and 7 show that the level of full credit between green and non-green companies is similar both before and after the COVID-19 pandemic, whereas significant differences exist for trade credit and short-term bank credit. In trade credit, green SMEs have a higher level than non-green SMEs, and in short-term bank credit, non-green SMEs have a higher level than green SMEs. Since green SMEs are more profitable both before and after the COVID-19 pandemic and the difference in trade credit level between green and non-green companies increases during the pandemic, trade credit might be considered as a component of a long-term portfolio management strategy, consolidating relationships with clients and increasing firm profitability in facing variable demand conditions. We also investigate a possible substitution effect between trade credit and short-term bank credit, using the main determinants of trade credit for green and non-green SMEs over the periods 2017–2020 and the two-year period 2019–2020 (see Table 8), according to OLS regression methodology (see Eq. 1). We also examine 2019 in comparison with 2020 (see Table 9), according to the diff-in-diff analysis (see Eq. 3). Our models include the following control variables: age, size, profitability, liquidity, trade credit in the previous year, account receivable, and short and long-term bank credit. The dependent variable is trade credit.

**Table 8:** Results of analysis—green and non-green SMEs—periods 2017–2020 and 2019–2020

Variables	Period 2017–2020		Period 2019–2020	
	Green	Non-green	Green	Non-green
Constant	0.165***	0.228***	0.0724**	0.0296***
	(0.033)	(0.007)	(0.0239)	(0.0034)
Age	− 0.013***	− 0.010***	− 0.0186***	− 0.0086***
	(0.003)	(0.001)	(0.0044)	(0.0006)
Size	− 0.002	− 0.014***	0.0056**	0.0037***
	(0.003)	(0.001)	(0.0024)	(0.0003)
Profitability	− 0.107***	− 0.027***	− 0.1166***	− 0.0383***
	(0.014)	(0.002)	(0.0164)	(0.0021)
Liquidity	− 0.027***	− 0.019***	− 0.0135***	− 0.0103***
	(0.002)	(0.001)	(0.0019)	(0.0003)
Trade credit_*t* − 1_	0.078***	0.081***	0.5088***	0.5707***
	(0.014)	(0.002)	(0.0141)	(0.0024)
Account receivable	0.269***	0.277***	0.1918***	0.1835***
	(0.011)	(0.002)	(0.0112)	(0.0020)
Short-term bank credit	− 0.151***	− 0.105***	− 0.0403***	− 0.0406***
	(0.021)	(0.003)	(0.0024)	(0.0025)
Long-term bank credit	− 0.055***	− 0.069***	0.0091	− 0.0007
	(0.021)	(0.003)	(0.0203)	(0.0028)
Year dummies	Included	Included	Included	Included
*R* squared	0.383	0.376	0.6618	0.7033

Robust standard errors are shown in brackets

*p-value < 0.1

**p-value < 0.05

***p-value < 0.01

**Table 9:** Results of analysis—green and non-green SMEs, 2019 in comparison with 2020

Variables	Green		Non-green	
	2019	2020	2019	2020
Constant	0.0288	0.0730**	0.0132**	0.0237***
	(0.0260)	(0.0259)	(0.0039)	(0.0037)
Age	− 0.0165**	− 0.0171***	− 0.0063***	− 0.0049***
	(0.0049)	(0.0045)	(0.0007)	(0.0006)
Size	0.0071*	− 0.0033	0.0037***	0.0014***
	(0.0026)	(0.0025)	(0.0004)	(0.0003)
Profitability	− 0.1219***	− 0.0941***	− 0.0594***	− 0.0252***
	(0.0197)	(0.0242)	(0.0027)	(0.0029)
Liquidity	− 0.0078**	− 0.0098***	− 0.00764***	− 0.0073***
	(0.0024)	(0.0020)	(0.0003)	(0.0002)
Trade credi_*t* − 1_	0.6751***	0.6510***	0.709***	0.6756***
	(0.0165)	(0.0176)	(0.0029)	(0.0029)
Account receivable	0.1411***	0.1157***	0.1210***	0.1334***
	(0.0128)	(0.0143)	(0.0024)	(0.0024)
Short-term bank credit	0.0260	− 0.0502*	0.0476***	− 0.0216***
	(0.0277)	(0.0301)	(0.0029)	(0.0035)
Long-term bank credit	0.0134	− 0.0036*	0.0215***	− 0.0157***
	(0.0257)	(0.0221)	(0.0039)	(0.0031)
Year dummies	Included	Included	Included	Included
*R* squared	0.6806	0.6730	0.7120	0.7146

Robust standard errors are shown in brackets. Dependent variable: trade credit

*p-value < 0.1

**p-value < 0.05

***p-value < 0.01

For trade credit, Table 8 shows:

- a negative relationship with age, liquidity, and particularly profitability and short-term bank credit for both green and non-green SMEs;
- a positive relationship with trade credi_t-1_, and with account receivable, which confirms the matching hypotheses, for both green and non-green SMEs.

The year-over-year comparison reported in Table 9 shows that during the COVID-19 pandemic a substitution effect between trade credit and short-term bank credit is verified, particularly for green companies.

## Conclusions and policy implications

This paper examines the financial resilience of green manufacturing compared to non-green manufacturing SMEs in Italy during the COVID-19 pandemic. Since the AIDA Bureau van Dijk database contains no specific data on green or ESG ratings, we consider as “green” those SMEs in industries characterized by higher green component than other industries, following the study by the Politecnico di Milano and Camera di Commercio di Milano (2012).

We verify the three hypotheses formulated in Sect. 2. As shown in Tables 6 and 7, the proportion of full credit is not higher for green firms than non-green firms both before and after the COVID-19 pandemic, so Hypothesis 1 is not proven. On the other hand, Hypotheses 2a and 2b are fully proven: the difference between green and non-green SMEs is shown in the importance of trade credit for the first group. Accounts payable and receivable are both significantly more important for green than for non-green SMEs, while short term bank credit is higher for non-green than for green SMEs (see Tables 6 and 7, 10, 11, 12). The overall analysis on green orientation and external credit shows that the COVID-19 pandemic affected the level and the differences in trade credit between green and non-green SMEs. Before the pandemic, green companies relied on a higher level of trade credit than non-green companies, and in 2020 the green company trade credit level stayed higher than the non-green company level. In other words, the difference in trade credit between green and non-green companies remained positive during the pandemic. This leads us to believe that a substitution effect between trade and short-term bank credit exists, in particular for green SMEs during the COVID-19 pandemic. In other words, trade credit received compensated for the reduction in short-term bank credit for green SMEs. Tables 8 and 9 in fact report that trade credit received is much more strongly negatively correlated to short-term bank credit for green SMEs than for non-green ones.

At the same time, descriptive statistics on the entire period as well as statistics for the years up to 2020, before the pandemic, show that, on average, green SMEs are younger and smaller but more profitable, and in better financial condition, than non-green SMEs. They also take up and offer more trade credit than non-green SMEs.

To conclude, the importance of trade credit for green SMEs could be explained by two complementary trends. A substitution effect, demonstrated by previous literature and recent research on Italian firms [Arcuri and Pisani (2021) on Italian green middle-sized companies] appears to occur on one segment of green SMEs (presumably the youngest/smallest and less profitable) not only in the pandemic period, but also before 2020. Meanwhile, for the segment of green SMEs which make average profitability of green SMEs higher than that of non-green SMEs, trade credit appears to be a component of long-term commercial strategy with clients (Emery 1987; Deloof et al. 1986) and for increasing firm profitability (Martínez-Sola and García-Tereul 2014), which, as noted above, is higher for green SMEs (Table 6). This ‘successful’ segment of green SMEs could be seen as a subclass of the green firms considered, in which trade credit is an important instrument of competition. This conclusion is in fact consistent with previous literature showing that the use of trade credit varies significantly across industries (Cannari et al. 2004; Ng et al. 1999; Marotta 2005; Wilson and Summers 2002).

## Data Availability

Our manuscript has no associate data.

## Descriptive statistics and analysis: further details

In Tables

**Table 10:** Descriptive statistics 2017–2019 in comparison with 2020

Variables	2017–2019		2020		*t* Statistic
	Mean	SE	Mean	SE	
Full credit	0.2845	0.0007	0.2375	0.0011	32.2411***
Trade credit	0.1816	0.0004	0.1546	0.0007	30.9286***
Profitability	0.0805	0.0004	0.0654	0.0007	17.7551***
Liquidity	1.4193	0.0033	1.7132	0.0069	− 41.4498***
Trade credit_*t* − 1_	0.1859	0.0004	0.1742	0.0007	13.2481***
Account receivable	0.2542	0.0005	0.2222	0.0008	29.8876***
Short-term bank credit	0.0906	0.0004	0.0640	0.0006	32.3858***
Long-term bank credit	0.0631	0.0003	0.0951	0.0006	− 46.4236***

**p*-value < 0.1

***p*-value < 0.05

****p*-value < 0.01

Source: AIDA Bureau van Dijk

10 and

**Table 11:** Descriptive statistics—green and non-green SMEs—Overall period (2017–2020)

Variables	Green		Non-green		*t* Statistic
	Mean	SE	Mean	SE	
Full credit	0.2778	0.0034	0.2727	0.0006	1.4348
Trade credit	0.1970	0.0023	0.1747	0.0003	10.7269***
Age	24.409	0.1599	29.704	0.0349	− 27.1432***
Size	8.6675	0.0143	8.5921	0.0025	5.4728***
Profitability	0.0876	0.0017	0.0767	0.0004	5.3969***
Liquidity	1.5760	0.0166	1.4835	0.0030	5.4526***
Trade credit_*t* − 1_	0.2031	0.0023	0.1823	0.0004	9.7335***
Account receivable	0.2901	0.0029	0.2454	0.0004	17.5092***
Short-term bank credit	0.0641	0.0016	0.0852	0.0003	− 10.7662***
Long-term bank credit	0.0706	0.0015	0.0705	0.0003	0.0551

**p*-value < 0.1

***p*-value < 0.05

****p*-value < 0.01

Source: AIDA Bureau van Dijk

11 further descriptive statistics are calculated. Table 10 shows the comparison between the three-year period 2017–2019 and 2020.

Table 11 reports the descriptive statistics of the two subsamples (green and non-green SMEs) for the whole period 2017–2020.

Tables

**Table 12:** Descriptive statistics for green and non-green SMEs, 2017–2019 in comparison with 2020

Variables	Green		Non-green			Green		Non-green		
	2017–2019					2020				
	Mean	SE	Mean	SE	*t* Statistic	Mean	SE	Mean	SE	*t* Statistic
Full credit	0.2920	0.0040	0.2843	0.0007	1.8022*	0.2351	0.0060	0.2375	0.0012	− 0.3638
Trade credit	0.2021	0.0026	0.1809	0.0004	8.7583***	0.1789	0.0044	0.1538	0.0007	6.4305***
Profitability	0.0805	0.0020	0.0803	0.0004	2.2486**	0.09471	0.0032	0.0645	0.0007	7.6590***
Liquidity	1.5257	0.0184	1.4157	0.0033	5.9559***	1.7527	0.0382	1.7119	0.0069	1.0287
Trade credit_*t* − 1_	0.2058	0.0027	0.1852	0.0004	8.2508***	0.1947	0.0045	0.1735	0.0007	5.2714***
Account receivable	0.2950	0.0034	0.2528	0.0005	14.2443***	0.2728	0.0057	0.2206	0.0008	10.6757***
Short-term bank credit	0.0674	0.0019	0.0913	0.0004	− 10.3746***	0.0526	0.0025	0.0644	0.0006	− 3.4356***
Long-term bank credit	0.0663	0.0016	0.0630	0.0003	1.7755*	0.0855	0.0033	0.0955	0.0006	− 2.7726*

**p*-value < 0.1

***p*-value < 0.05

****p*-value < 0.01

Source: AIDA Bureau van Dijk

12 summarizes descriptive statistics over the sub-periods 2017–2019 and 2020.

Table

**Table 13:** Results of diff-in-diff analysis (dependent variables: full credit, Trade credit, Short-term bank credit, Long-term bank credit)

	Dependent variable (outcome variables)			
	Full credit	Trade credit	Short-term bank credit	Long-term bank credit
Constant	0.2157***	0.2170***	0.0088**	− 0.0158***
	(0.0061)	(0.0033)	(0.0031)	(0.0026)
Green	0.0033	0.01764***	− 0.0190***	0.0053**
	(0.0041)	(0.0023)	(0.0021)	(0.0018)
COVID	− 0.0083***	− 0.0213***	− 0.0214***	0.0347***
	(0.0016)	(0.0009)	(0.0008)	(0.0007)
Green*COVID	− 0.0013	0.0041**	0.0120**	− 0.0145***
	(0.0088)	(0.0018)	(0.0045)	(0.0038)
Age	− 0.0214***	− 0.0354***	0.0107***	0.0028***
	(0.0011)	(0.0006)	(0.0006)	(0.0005)
Size	0.0357***	0.0141***	0.0118***	0.0104***
	(0.0007)	(0.0004)	(0.0003)	(0.0003)
Profitability	− 0.1406***	− 0.0022	− 0.1036***	− 0.0258***
	(0.0044)	(0.0024)	(0.0023)	(0.0019)
Liquidity	− 0.0713***	− 0.0275***	− 0.0313***	− 0.0123***
	(0.0005)	(0.0003)	(0.0003)	(0.0002)
N. observations	175,618	175,480	175,605	175,618
*R* squared	0.1225	0.0826	0.1053	0.0392

Robust standard errors are shown in brackets

**p*-value < 0.1

***p*-value < 0.05

****p*-value < 0.01

^13^ shows the results of DID analysis considering 2017–2019 as the period before the treatment (i.e., 2020).

Table

**Table 14:** Results of analysis—green and non-green SMEs, 2017–2019 in comparison with 2020

Variables	Green		Non-green	
	2017–2019	2020	2017–2019	2020
Constant	0.0369**	0.0730**	0.0169***	0.0237***
	(0.0151)	(0.0259)	(0.0023)	(0.0037)
Age	− 0.0065**	− 0.0171***	− 0.0073***	− 0.0049***
	(0.0029)	(0.0045)	(0.0004)	(0.0006)
Size	− 0.0035**	− 0.0033	− 0.0038***	− 0.0014***
	(0.003)	(0.0025)	(0.0002)	(0.0003)
Profitability	− 0.1309***	− 0.0941***	− 0.0492***	− 0.0252***
	(0.0129)	(0.0242)	(0.0016)	(0.0029)
Liquidity	− 0.0099***	− 0.0098***	− 0.0073***	− 0.0073***
	(0.0013)	(0.0020)	(0.0002)	(0.0002)
Trade credi_*t* − 1_	0.697***	0.6510***	0.697***	0.6756***
	(0.0096)	(0.0176)	(0.0017)	(0.0029)
Account receivable	0.1299***	0.1157***	0.1358***	0.1334***
	(0.0075)	(0.0143)	(0.0014)	(0.0024)
Short-term bank credit	− 0.0486***	− 0.0502*	− 0.0252***	− 0.0216***
	(0.0142)	(0.0301)	(0.0017)	(0.0035)
Long-term bank credit	0.0078	− 0.0036*	0.0276***	− 0.0157***
	(0.0148)	(0.0221)	(0.0022)	(0.0031)
Year dummies	Included	Included	Included	Included
*R* squared	0.6822	0.6730	0.7005	0.7146

Robust standard errors are shown in brackets. Dependent variable: trade credit

**p*-value < 0.1

***p*-value < 0.05

****p*-value < 0.01

^14^ reports results of analysis on the main determinants of trade credit for green and non-green SMEs for the sub-period 2017–2019 compared with 2020. The model includes the following control variables: age, size, profitability, liquidity, trade credit in the previous year, account receivable, and short and long-term bank credit. The dependent variable is trade credit.

In Tables

**Table 15:** Results of analysis – Green and non-green SMEs, overall period (2017–2020) in comparison with 2019–2020– (Dependent variable: Full credit)

Variables	Period 2017–2020		Period 2019–2020	
	Green	Non-green	Green	Non-green
Constant	0.05351**	− 0.0409***	0.0891*	0.051*
	(0.0230)	(0.0041)	(0.0538)	(0.0102)
Age	0.0028	− 0.0012	0.0071	− 0.0038**
	(0.0044)	(0.0007)	(0.0101)	(0.0019)
Size	0.0080**	0.0169***	0.0175**	0.0272***
	(0.0023)	(0.0004)	(0.0053)	(0.0010)
Profitability	− 0.2316***	− 0.1604***	− 0.1900***	− 0.1898***
	(0.0185)	(0.0029)	(0.0276)	(0.0045)
Liquidity	− 0.0262***	− 0.0266***	− 0.0407***	− 0.0373***
	(0.0020)	(0.0003)	(0.0035)	(0.0006)
Trade credi_*t* − 1_	0.6723***	0.7174***	0.3183***	0.4096***
	(0.0148)	(0.0030)	(0.0262)	(0.0059)
Account receivable	0.1629***	0.2461***	0.2816***	0.4066***
	(0.0116)	(0.0025)	(0.0211)	(0.0049)
Year dummies	Included	Included	Included	Included
*R* squared	0.6041	0.6427	0.3536	0.3846

Robust standard errors are shown in brackets

**p*-value < 0.1

***p*-value < 0.05

****p*-value < 0.01

^15^ and

**Table 16:** Results of analysis—green and non-green SMEs, 2017–2019 in comparison with 2020 (dependent variable: full credit)

Variables	Green		Non-green	
	2017–2019	2020	2017–2019	2020
Constant	0.0166*	0.1453***	− 0.0685***	0.03737***
	(0.0274)	(0.0375)	(0.0049)	(0.0011)
Age	0.0099*	− 0.0246***	0.0005	− 0.0115***
	(0.0054)	(0.0065)	(0.0009)	(0.0011)
Size	0.0109***	0.0024	0.0211***	0.0056***
	(0.0027)	(0.0038)	(0.0005)	(0.0007)
Profitability	− 0.2424***	− 0.1593***	− 0.1655***	− 0.1550***
	(0.0213)	(0.0352)	(0.0034)	(0.0051)
Liquidity	− 0.0288***	− 0.0151***	− 0.0311***	− 0.0089***
	(0.0024)	(0.0029)	(0.0004)	(0.0005)
Trade credi_*t* − 1_	0.6832***	0.6172***	0.7183***	0.7005***
	(0.0175)	(0.0252)	(0.0035)	(0.0053)
Account receivable	0.1610***	0.1612***	0.2529***	0.1824***
	(0.0136)	(0.0207)	(0.0029)	(0.0044)
Short-term bank credit_*t* − 1_	0.7662***	0.7171***	0.6637***	0.8436***
	(0.0288)	(0.0386)	(0.0039)	(0.0055)
Long-term bank credit_*t* − 1_	0.800***	0.8659***	0.8145***	0.8502***
	(0.0302)	(0.0362)	(0.0049)	(0.0072)
Year dummies	Included	Included	Included	Included
*R* squared	0.5904	0.7001	0.6233	0.7450

Robust standard errors are shown in brackets. Dependent variable: full credit. We add the control variables Short-term bank credit_*t* − 1_ and long-term bank credit_*t* − 1_

**p*-value < 0.1

***p*-value < 0.05

****p*-value < 0.01

^16^ we present further analysis showing the main determinants of full credit for green and non-green SMEs over the overall period (2017–2020) and the two-year period 2019–2020, distinguishing between green and non-green SMEs. Our models include the following control variables: age, size, profitability, liquidity, trade credit in the previous year, account receivable, and short and long-term bank credit.

## Footnotes

1 The EU defines a “green economy” as an “economy that can secure growth and development, while at the same time improving human well-being, providing decent jobs, reducing inequalities, tackling poverty and preserving the natural capital upon which we all depend”. European Commission “Rio + 20: towards the green economy and better performance”, Communication 2011.

2 See: https://ec.europa.eu/growth/smes/sme-definition_it.

3 The variable “Full credit” is calculated for each SME as the sum between bank credit and trade credit divided by total assets. The mean of this variable is calculated considering the value for all SMEs over the entire period.

4 This special measure provided for state guarantees for bank loans made to companies and the self-employed for amounts related to turnover in previous years and for a duration of up to 6 years, on very favourable conditions.

5 From 2011 to 2018 bank loans to companies decreased on average by 4.74% per year. In 2020 bank loans to companies increased by 5.6%.

## References

[CR1] Abu Bakar LJ (2011) Relationship between firm resources and product innovation performance in Malaysian small medium enterprises: the moderating role of age and size. PhD thesis, Sintok, Kedah, University Utara Malaysia

[CR2] Alessandrini P, Presbitero AF, Zazzaro A (2009). Banks, distances and firms’ financing constraints. Rev Financ.

[CR3] Arcuri MC, Pisani R (2021). Is trade credit a sustainable resource for medium-sized Italian Green companies?. Sustainability.

[CR4] Ayyagari M, Demirguc-Kunt A, Maksimovic V (2011) Small vs. young firms across the world: contribution to employment, job creation, and growth. Policy research working paper n. WPS 5631, World Bank

[CR5] Baas T, Schrooten M (2006). Relationship banking and SMEs: a theoretical analysis. Small Bus Econ.

[CR6] Banerjee R, Gupta K, McIver R (2019). What matters most to firm-level environmentally sustainable practices: firm–specific or country–level factors?. J Clean Prod.

[CR7] Barbier EB (2012). The green economy post Rio+20. Science.

[CR8] Bartik AW, Bertrand M, Cullen ZB, Glaeser EL, Luca M, Stanton CT (2020) How are small businesses adjusting to covid-19? Early evidence from a survey. National Bureau of Economic Research.

[CR9] Bartz W, Winkler A (2016). Flexible or fragile? The growth performance of small and young businesses during the global financial crisis—evidence from Germany. J Bus Ventur.

[CR10] Bastos R, Pindado J (2013). Trade credit during a financial crisis: a panel data analysis. J Bus Res.

[CR11] Battisti M, Deakins D (2012) Perspectives from New Zealand small firms: Crisis management and the impact of the Canterbury earthquakes. In: New Zealand Centre for Small and Medium Enterprise Research (ed.) BusinesSMEasure. Massey University, pp 1–41

[CR12] Bauer R, Hann D (2010). Corporate Environmental Management and Credit Risk.

[CR13] Beck T, Demirguc-Kunt A (2006). Small and medium-size enterprises: access to finance as a growth constraint. J Bank Finance.

[CR14] Benfratello L, Schiantarelli F, Sembenelli A (2008). Banks and innovation: microeconometric evidence on Italian firms. J Financ Econ.

[CR15] Berg F, Kölbel JF, Rigobon R (2022). Aggregate confusion: the divergence of ESG ratings. Rev Financ.

[CR16] Berger AN, Udell GF (1998). The economics of small business finance: the roles of private equity and debt markets in the financial growth cycle. J Bank Financ.

[CR17] Bertrand M, Duflo E, Mullainathan S (2004). How much should we trust differences-in-differences estimates?. Q J Econ.

[CR18] Biais B, Gollier C (1997). Trade credit and credit rationing. Rev Financ Stud.

[CR19] Bonaccorsi di Patti E, Gobbi G (2001). The changing structure of local credit markets: Are small business special?. J Bank Finance.

[CR20] Bougheas S, Mateut S, Mizen P (2009). Corporate trade credit and inventories: new evidence of a trade-off from account payable and receivable. J Bank Finance.

[CR21] Bouslah K, Kryzanowski L, M’Zali B (2018). Social performance and firm risk: impact of the financial crisis. J Bus Ethics.

[CR22] Bragdon J, Marlin J (1972). Is pollution profitable?. Risk Manage.

[CR23] Branco MC, Rodrigues LL (2006). Corporate social responsibility and resource-based perspectives. J Bus Ethics.

[CR24] Brechling F, Lipsey R (1963). Trade credit and monetary policy. Econ J.

[CR25] Brennan M, Maksimovic V, Zechner J (1988). Vendor financing. J Financ.

[CR26] Brown R, Rocha A (2020). Entrepreneurial uncertainty during the Covid-19 crisis: mapping the temporal dynamics of entrepreneurial finance. J Bus Ventur Insights.

[CR27] Bruna MG, Nicolo D (2020). Corporate reputation and social sustainability in the early stages of start-ups: a theoretical model to match stakeholders’ expectations through corporate social commitment. Financ Res Lett.

[CR28] Bukart M, Ellingsen T (2004). In-kind finance: a theory of trade. Am Econ Rev.

[CR29] Burns P (2016) Entrepreneurship and small business. Palgrave

[CR30] Bussoli C (2017). Trade credit financing: substitution and matching effect for Italian SMEs. J Econ Finance Adm Sci.

[CR31] Cannari L, Chiri S, Omiccioli M (2004) Condizioni di credito commerciale e differenziazione della clientela. Temi di Discussione del Servizio Studi, Banca d’Italia

[CR32] Canto-Cuevas FJ, Palacín-Sánchez MJ, Di Pietro F (2019). Trade credit as a sustainable resource during an SME’s life cycle. Sustainability.

[CR33] Canto-Cuevas FJ, Palacín-Sánchez MJ, Di Pietro F (2016). Trade credit in SMEs: a quantile regression approach. Appl Econ Lett.

[CR34] Caprotti F, Bailey I (2014). Making sense of the green economy. Geogr Ann Ser B.

[CR35] Carbo-Valverde S, Rodriguez-Fernandez F, Udell GF (2016). Trade credit, the financial crisis, and SME access to finance. J Money Credit Bank.

[CR36] Casey E, O’Toole CM (2014). Bank lending constraints, trade credit and alternative financing during the financial crisis: evidence from European SMEs. J Corp Finance.

[CR37] Choi WG, Yungsan K (2005). Trade credit and the effect of macro-financial shocks: evidence from U.S. panel data. J Financ Quant Anal.

[CR38] Cull R, Xu LC, Zhu T (2009). Formal finance and trade credit during China’s transition. J Financ Intermed.

[CR39] Cuñat V (2007). Trade credit: suppliers as debt collectors and insurance providers. Rev Financ Stud.

[CR40] D’Amato D (2021) Sustainability narratives as transformative solution pathways: zooming in on the circular economy. Circ Econ Sustain. 10.1007/s43615-021-00008-1

[CR41] D’Amato D, Korhonen J (2021). Integrating the green economy, circular economy and bioeconomy in a strategic sustainability framework. Ecol Econ.

[CR42] Davidson P, Gordon S (2016). Much ado about nothing? The surprising persistence of nascent entrepreneurs through macroeconomic crisis. Entrep Theory Pract.

[CR43] Dedola L, Lippi F (2000) The monetary transmission mechanism: evidence from the industries of five OECD countries. Temi di discussione, n. 389, Banca d’Italia

[CR44] Deloof M, Jegers M (1996). Trade credit, product quality, and intragroup trade: some European evidence. Financ Manage.

[CR45] Demirguc-Kunt A, Lokshin M, Torre I (2021). The sooner, the better: the economic impact of non-pharmaceutical interventions during the early stage of the COVID19 pandemic. Econ Trans Inst Chang.

[CR46] Demirgüç-Kunt A, Maksimovic V (2001) Firms as financial intermediaries. Evidence from trade credit data. Policy research working paper, World Bank Development Research Group, WP 2696

[CR47] Doern R, Williams N, Vorley T (2019). Special issue on entrepreneurship and crises: Business as usual? An introduction and review of the literature. Entrep Reg Dev.

[CR48] Donthu N, Gustafson A (2020). Effects of COVID-19 on business and research. J Bus Res.

[CR49] Duca J (1986). Trade credit and credit rationing: a theoretical model.

[CR50] Emery GW (1987). An optimal financial response to variable demand. J Financ Quant Anal.

[CR51] European Commission (2018) The inclusive green economy in EU development cooperation. An innovative approach at the intersection of the EU’s Planet, People and Prosperity objectives. Tools and Methods series, Reference document n. 25, 2018. https://op.europa.eu/en/publication-detail/-/publication/a7a02150-01ad-11e9-adde-01aa75ed71a1

[CR52] European Commission (2019) SBA Fact Sheet Italy. European Union, Luxembourg

[CR53] Fabbri D, Klapper L (2008) Market power and the matching of trade credit terms. World Bank Policy research working paper, n. 4754

[CR54] Farhoud M, Shah S, Stenholm P, Kibler E, Renko M, Terjesenf S (2021). Social enterprise crowdfunding in an acute crisis. J Bus Ventur Insights.

[CR55] Farza K, Ftiti Z, Hlioui Z, Louhichi W, Omri A (2021). Does it pay to go green? Environmental innovation effect on corporate financial performance. J Environ Manag.

[CR56] Fisman R, Love I (2003). Trade credit, financial intermediary development, and industry growth. J Financ.

[CR57] Fort T, Haltiwanger J, Jarmin R, Miranda J (2012) How firms respond to business cycles: the role of the firm age and firm size. In: 13th Jacques Polak annual research conference, international monetary fund Washington, November 8–9, pp 1–65

[CR58] Freixas X (1993) Short term credit versus account receivable financing. Working paper, Universitat Pompeu Fabra

[CR59] Fukuda SI, Kasuya M, Akashi K (2007). The role of trade credit for small firms: an implication from Japan’s banking crisis. J Public Policy Rev.

[CR60] Gagliardi F (2009). Financial development and the growth of cooperative firms. Small Bus Econ.

[CR61] Garcia-Appendini E, Montoriol-Garriga J (2013). Firms as liquidity providers: evidence from the 2007–2008 financial crisis. J Financ Econ.

[CR62] García-Carbonell N, Martín-Alcázar F, Sánchez-Gardey G (2021). Facing crisis periods: a proposal for an integrative model of environmental scanning and strategic issue diagnosis. Rev Manag Sci.

[CR63] García-Teruel PJ, Martínez-Solano P (2010). Determinants of trade credit: a comparative study of European SMEs. Int Small Bus J.

[CR64] Gilmore A, McAuley A, Gallagher D, Massiera P, Gamble J (2013). Researching SME/ entrepreneurial research. J Res Mark Entrep.

[CR65] Godfrey PC (2005). The relationship between corporate philanthropy and shareholder wealth: a risk management perspective. Acad Manag Rev.

[CR66] Godfrey PC, Merrill CB, Hansen JM (2009). The relationship between corporate social responsibility and shareholder value: an empirical test of the risk management hypothesis. Strateg Manag J.

[CR67] Goss A, Roberts GS (2011). The impact of corporate social responsibility on the cost of bank loans. J Bank Finance.

[CR68] Guerin P, Suntheim F (2021). Firms’ environmental performance and the COVID-19 crisis. Econ Lett.

[CR69] Guiso L, Kashyap AK, Panetta F, Terlizzese D (2000) Will a common European Monetary Policy have asymmetric effects? Temi di discussione, N. 384, Banca d’Italia

[CR70] Guiso L, Sapienza P, Zingales L (2004). Does local financial development matter?. Q J Econ.

[CR71] Hong H, Liskovich I (2019) Crime, punishment and the halo effect of corporate social responsibility. https://ceep.columbia.edu/sites/default/files/content/events/Halo_v14.pdf

[CR72] Huang W, Chen S, Nguyen LT (2020). Corporate social responsibility and organizational resilience to COVID-19 crisis: an empirical study of Chinese firms. Sustainability.

[CR73] International Trade Centre (2020). SME competitiveness outlook 2020: COVID-19: the great lockdown and its impact on small business.

[CR74] Herbane B (2013). Exploring crisis management in UK small- and medium-sized enterprises. J Contingenc Crisis Manag.

[CR75] Herbane B (2019). Rethinking organizational resilience and strategic renewal in SMEs. Entrep Reg Dev.

[CR76] Herbst AF (1974). Some empirical evidence on the determinants of trade credit at the industry level of aggregation. J Financ Quant Anal.

[CR77] Jaffee DM (1971). Credit rationing and the commercial loan market.

[CR78] Jaffee DM, Modigliani F (1969). A theory and test of credit rationing. Am Econ Rev.

[CR79] Jain N (2001). Monitoring costs and trade credit. Q Rev Econ Financ.

[CR80] Jo H, Na H (2012). Does CSR reduce firm risk? Evidence from controversial industry sectors. J Bus Ethics.

[CR81] Jordan J, Lowe J, Taylor P (1998). Strategy and financial policy in UK small firms. J Bus Financ Acc.

[CR82] Juergensen J, Guimón J, Narula R (2020). European SMEs amidst the COVID-19 crisis: assessing impact and policy responses. J Ind Bus Econ.

[CR83] Kang SJ, Lee S (2021). Impacts of environmental policies on global green trade. Sustainability.

[CR84] Kasztelan A (2017). Green growth green economy and sustainable development: terminological and relational discourse. Prague Econ. Pap..

[CR85] Kiernan MJ (2007). Universal Owners and ESG: leaving money on the table?. Corp Gov: Int Rev.

[CR86] Korhonen J, Granberg B (2020). Sweden backcasting, now? Strategic planning for Covid-19 mitigation in a liber democracy. Sustainability.

[CR87] Kuckertz A, Brandle L, Gaudig A, Hinderer S, Reyes CAM, Prochotta A, Berger ES (2020) Startups in times of crisis—a rapid response to the COVID-19 pandemic. J Bus Ventur Insights 13:e00169. 10.1016/j.jbvi.2020.e00169

[CR88] Lahouel BB, Bruna M-G, Zaied YB (2020). The curvilinear relationship between environmental performance and financial performance: an investigation of listed french firms using panel smooth transition model. Financ Res Lett.

[CR89] La Rocca M, La Rocca T, Cariola A (2010). The influence of local institutional differences on the capital structure of SMEs: evidence from Italy. Int Small Bus J.

[CR90] Lavrinenko O, Ignatjeva S, Ohotina A, Rybalkin O, Lazdans D (2019). The role of green economy in sustainable development (Case study: The EU States). Entrep Sustain Issues.

[CR91] Lee D, Faff R (2009). Corporate sustainability performance and idiosyncratic risk: a global perspective. Financ Rev.

[CR93] Lee YW, Stowe JD (1993). Product risk, asymmetric information, and trade credit. J Financ Quant Anal.

[CR94] Howell S, Lerner J, Nanda R, Townsend R (2020) Financial distancing: how venture capital follows the economy down and curtails innovation. Harvard Business School Working Papers, Harvard Business School, pp 20–115

[CR95] Lins KV, Servaes H, Tamayo A (2017). Social capital, trust, and firm performance: the value of corporate social responsibility during the financial crisis. J Financ.

[CR96] Lins KV, Servaes H, Tamayo A (2019). Social capital, trust, and corporate performance: how CSR helped companies during the financial crisis (and why it can keep helping them). J Appl Corp Financ.

[CR97] Long MS, Malitz IB, Ravid SA (1993). Trade credit, quality guarantees, and product marketability. Financ Manage.

[CR98] Love I, Preve LA, Sarria-Allende V (2007). Trade credit and bank credit: evidence from recent financial crises. J Financ Econ.

[CR99] Magnanelli BS, Izzo MF (2017). Corporate social performance and cost of debt: the relationship. Soc Responsib J.

[CR100] Marotta G (2005). When do trade credit discounts matter? Evidence from Italian firm-level data. Appl Econ.

[CR101] Marsat S, Pijourlet G, Ullah M (2020). Is there a trade-off between environmental performance and financial resilience? International evidence from the subprime crisis. Acc Financ.

[CR102] Martin D, Romero I, Wegner D (2019). Individual, organizational, and institutional determinants of formal and informal inter-firm cooperation in SMEs. J Small Bus Manage.

[CR103] Martínez-Sola C, García-Tereul PJ (2014). Trade credit and SME profitability. Small Bus Econ.

[CR104] Mateut S (2005). Trade credit and monetary policy transmission. J Econ Surv.

[CR105] Mateut S, Bougheas S, Mizen P (2006). Trade credit, bank lending and monetary policy transmission. Eur Econ Rev.

[CR106] Matias Gama AP, Van Auken H (2015). The interdependence between trade credit and bank lending: commitment in intermediary firm relationships. J Small Bus Manag.

[CR107] May S, Lixl D (2019). Restructuring in SMEs—a multiple case study analysis. J Small Bus Strateg.

[CR108] McGuinness G, Hogan T (2014). Bank credit and trade credit: evidence from SMEs over the financial crisis. Int Small Bus J.

[CR109] McMillan J, Woodruff C (1999). Interfirm relationships and informal credit in Vietnam. Q J Econ.

[CR110] Meltzer AH (1960). Mercantile credit, monetary policy and the size of firms. Rev Econ Stat.

[CR111] Mian SL, Smith CW (1994). Extending trade credit and financing receivables. J Appl Corp Financ.

[CR112] Mol APJ, Sonnenfeld DA (2000). Ecological modernisation around the world: an introduction. Environ Polit.

[CR113] Muhammad N, Scrimgeour F, Reddy K, Abidin S (2015). The impact of corporate environmental performance on market risk: the Australian industry case. J Bus Ethics.

[CR114] Nilsen J (2002). Trade credit and the bank lending channel. J Money Credit Bank.

[CR115] Ng CK, Smith JK, Smith RLE (1999). vidence on the determinants of credit terms used in interfirm trade. J Finance.

[CR116] O'Callaghan B, Murdock E (2021) Are we building back better? Evidence from 2020 and pathways for inclusive green recovery spending. Oxford University Economic Recovery Project, United Nations Environment Programme

[CR117] OECD (2020) Issue Note 2: Corporate sector vulnerabilities during the COVID-19 outbreak: assessment and policy responses. In: OECD Economic Outlook 2020(1), OECD Publishing, Paris. 10.1787/6434b1e4-en

[CR118] Ogawa K, Sterken E, Tokutsu I (2013). The trade credit channel revisited: evidence from micro data of Japanese small firms. Small Bus Econ.

[CR119] Palahí M, Pantsar, M, Costanza R, Kubiszewski I, Potočnik J, Stuchtey M, Nasi R, Lovins H, Giovannini E, Fioramonti L, Dixson-Declève S, McGlade J, Pickett K, Wilkinson R, Holmgren J, Trebeck K, Wallis S, Ramage M, Berndes G, Akinnifesi FK, Ragnarsdóttir KV, Muys B, Safonov G, Nobre AD, Nobre C, Ibañez D, Wijkman A, Snape J, Bas L (2020) Investing in Nature as the true engine of our economy: a 10-point action plan for a circular bioeconomy of wellbeing. Knowledge to Action 02, European Forest Institute

[CR120] Paul S, Wilson N (2007). The determinants of trade credit demand: Survey evidence and empirical analysis. J Small Bus Manag.

[CR121] Petersen M, Rajan R (1997). Trade credit: theories and evidence. Rev Financ Stud.

[CR122] Pike R, Cheng NS, Cravens K (2005). Trade credits terms: asymmetric information and price discrimination evidence from three continents. J Bus Financ Acc.

[CR123] Politecnico di Milano; Camera di Commercio di Milano (2012) Analisi dei mercati e delle filiere “green” in Lombardia, Vittorio Chiesa, Eds

[CR124] Porter ME, Van der Linde C (1995). Toward a new conception of the environment-competitiveness relationship. J Econ Perspect.

[CR125] PricewaterhouseCoopers (2013) 16th CEO survey. http://www.pwc.com/gx/en/ceo-survey/2013/assets/pwc-16th-global-ceo-survey_jan-2013.pdf

[CR126] Psillaki M, Eleftheriou K (2015). Trade credit, bank credit, and flight to quality: evidence from French SMEs. J Small Bus Manag.

[CR128] Schwartz RA, Whitcomb DK, Bounding E, Wilson TF (1978). Implicit transfers in the extension of trade credit. Redistribution through the financial system: the grants economics of money and credit.

[CR127] Schwartz RA, Whitcomb D, Bicksler JL (1979). The trade credit decision. Handbook of financial economics.

[CR130] Seeger MW, Hipfel SJ (2007) Legal versus ethical arguments: contexts for corporate social responsibility. In: May S, Cheney G, Roper J (ed) The debate over corporate social responsibility, pp 155–167

[CR131] Sharfman MP, Fernando CS (2008). Environmental risk management and the cost of capital. Strateg Manag J.

[CR132] Šipilova V, Ostrovska I, Jermolajeva E, Aleksejeva L, Oļehnovičs D (2017). Evaluation of sustainable development in rural territories in Latgale region (Latvia) by using the conception of smart specialization. J Teach Edu Sust.

[CR134] Taherzadeh O (2021). Promise of a green economic recovery postCovid: Trojan horse or turning point?. Glob Sustain.

[CR135] Taketa K, Udell GF (2007). Lending channels and financial shocks: the case of small and medium-sized enterprise trade credit and the Japanese banking crisis. Monet Econ Stud.

[CR136] Tsuruta D (2015). Bank loan availability and trade credit for small businesses during the financial crisis. Q Rev Econ Financ.

[CR137] UNEPP (2011) Towards a green economy: pathways to sustainable development and poverty eradication. UNEPP Report

[CR138] Van Der Wijst N, Thurik R (1993). Determinants of small firm debt ratios: an analysis of retail panel data. Small Bus Econ.

[CR139] Yang X (2011). The role of trade credit in the recent subprime financial crisis. J Econ Bus.

[CR140] Yang X, Zhang X, Lee PK (2019). Improving the effectiveness of online healthcare platforms: an empirical study with multi-period patient doctor consultation data. Int J Prod Econ.

[CR141] Yazdanfar D, Öhman P (2017). Substitute or complement? The use of trade credit as a financing source among SMEs. Manag Res Rev.

[CR142] Wellalage HN, Kumar V (2020) Does it pay to be green? Environmental performance and firm financing during COVID-19 outbreaks. Research Square 10.1016/j.frl.2021.102568 PMC8608384 34840534

[CR143] Wellalage NH, Kumar V (2021). Environmental performance and bank lending: evidence from unlisted firms. Bus Strateg Envirn.

[CR144] Wellalage HN, Kumar V, Hunjra AI, Al-Faryan M-F (2021). Environmental performance and firm financing during COVID-19 outbreaks: evidence from SMEs. Financ Res Lett.

[CR145] Wilner BS (2007). The exploitation of relationship in financial distress: the case of trade credit. J Financ.

[CR146] World Bank (2012). Inclusive green growth: the pathway to sustainable development.

[CR147] Zeidan R, Boechat C, Fleury A (2015). Developing a sustainability credit score system. J Bus Ethics.

